# The impact of using suture ligatures as a replacement for staplers and reducing excessive thermal energy on outcomes after pulmonary segmentectomy: a prospective cohort study

**DOI:** 10.3389/fonc.2025.1628701

**Published:** 2025-10-23

**Authors:** Binkui Wang, Fangqing Wang, Gang Chen, Weimin Ruan, Zhaowang Zhu, Weijian Hu, Lin Zang

**Affiliations:** Department of Cardiothoracic Surgery, The People’s Hospital of Tongling, Tongling, Anhui, China

**Keywords:** thoracoscopy, lung cancer, lung segmentectomy, postoperative cough, bronchial protection, ultrasonic scalpel

## Abstract

**Background:**

This study aims to evaluate the impact of using suture ligatures and avoiding thermal injury (hereinafter referred to as the modified measures) on clinical outcomes after pulmonary segmentectomy.

**Methods:**

A prospective randomized controlled study was conducted, involving 100 patients who met the inclusion criteria and were scheduled for single-port video-assisted thoracoscopic lung segmentectomy at our hospital from May 1, 2024, to May 31, 2025. The patients were randomly divided into an experimental group (modified measures) and a control group (traditional lung segmentectomy), followed by undergoing single-port thoracoscopic lung segmentectomy. A questionnaire was used to evaluate the incidence of cough and the intensity of cough symptoms at 2, 4, and 8 weeks postoperatively, along with related intraoperative and postoperative indicators, to verify the effectiveness and safety of the improved intraoperative bronchial protection measures in single-port thoracoscopic lung segmentectomy.

**Results:**

The modified measures significantly reduced the incidence of cough and symptom intensity at 2, 4, and 8 weeks postoperative. The cough scores in the experimental group were significantly better than those in the control group, with a statistically significant difference (p < 0.05). Analysis of intraoperative and postoperative indicators showed that the improved measures did not increase the surgical time and that the total intraoperative blood loss and the total drainage volume on postoperative day 3 were similar between the two groups, with no statistically significant difference (p > 0.05).

**Conclusion:**

Using suture ligatures as a replacement for staplers and minimizing thermal injury to the hilar structures based on standardized perioperative management may positively impact postoperative clinical outcomes, particularly the severity and duration of postoperative cough, leading to a significant improvement in patients’ quality of life.

## 1 Introduction

Thoracoscopic lung segmentectomy, as an important development in minimally invasive surgical technology, has been widely applied in the treatment of early-stage lung cancer, lung metastases, and benign pulmonary lesions (1, 2). This surgical technique achieves the goal of radical resection while preserving the patient’s lung function to the greatest extent, embodying the core concept of modern precision medicine (3). Postoperative cough is a common complication of this surgery, which not only severely affects the patient’s postoperative quality of life but also increases the incidence of surgical complications. How to reduce postoperative cough has become an urgent issue for clinicians to address (4).

Surgical operation is one of the key causes of postoperative cough, and its pathophysiological mechanisms are mainly related to mechanical injury of the bronchus, disruption of neuronal pathways during the operation, and postoperative inflammatory responses (5, 6). Traditional thoracoscopic lung segmentectomy uses a stapling device to handle the bronchus, which, although convenient, often causes bronchial tearing and the retention of metal foreign bodies at the bronchial stump. At the same time, thermal damage caused by the use of an electrocoagulation hook during the dissection of the bronchus and surrounding tissues can also damage the bronchial mucosa and surrounding neuronal pathways, thereby worsening postoperative cough symptoms. Clinical data show that the incidence of acute cough after lung segmentectomy can be as high as 50%-70%, with some patients experiencing cough symptoms for several weeks or even months, and the incidence of chronic cough can reach 30%-40%, significantly affecting their postoperative quality of life (7, 8).

Based on the aforementioned mechanisms of postoperative cough, our research team focuses on the concept of bronchial protection in single-port thoracoscopic lung segmentectomy and adopts a series of bronchial protection measures, including using silk sutures to ligate the bronchial stump instead of using a stapling device and using an ultrasonic scalpel to dissect and free the bronchus instead of using an electrocoagulation hook. These improvements significantly reduce mechanical and thermal damage to the bronchus and are expected to lower the incidence of postoperative cough by optimizing surgical techniques, shorten the patient’s recovery time, and improve the overall therapeutic effect of the surgery. This study aims to demonstrate the effectiveness and safety of these improved measures.

## 2 Materials and methods

### 2.1 General information

A total of 100 patients scheduled to undergo single-port thoracoscopic lung segmentectomy from May 1, 2024, to May 31, 2025, were selected. Inclusion criteria: 1) Chest CT showed peripheral lung nodules with a long diameter ≤ 2 cm, with lung cancer not being excluded; 2) Preoperative discussion and assessment indicated suitability for single lung segment resection; 3) Age ≥ 20 years and ≤ 80 years, regardless of gender; 4) Complete clinical data. Exclusion criteria: 1) Coexisting chronic cough symptoms such as chronic bronchitis, emphysema, bronchial asthma, postnasal drip syndrome, or those using angiotensin-converting enzyme inhibitors; 2) Upper respiratory tract infection, lung infection, or other preoperative cough symptoms within 2 weeks before surgery; 3) Coexisting severe heart, lung, liver, or kidney dysfunction, or other underlying diseases such as other malignancies; 4) Lost to follow-up or patients unable to cooperate.

### 2.2 Sample size and case allocation

The project team conducted an extensive literature review to understand the range of sample sizes in similar studies. We found that, although sample sizes varied across different research, many studies focused on a range of 80 to 120 cases. Based on this, we selected 100 cases as the preliminary sample size to ensure that our data had sufficient statistical significance. At the same time, we took into account practical feasibility, including patient recruitment speed, research duration, and available resources. We expect that recruiting 100 eligible patients during the study period is feasible; even with a certain proportion of loss to follow-up or missing data, this sample size can still support effective data analysis. We used G*power software to calculate the required sample size, with a target effect size of 0.8 (large effect), a significance level of α=0.01, and a test power of 0.8, which means that at least 39 cases are needed per group. After considering the research results and inclusion/exclusion criteria, we finally determined that each group would consist of 50 cases. This study utilized a central randomization method; prior to surgery, patients were randomly assigned to the modified bronchial protection measures group (experimental group) and the traditional pulmonary segment resection group (control group) based on a random number table generated by a computer, with 50 cases in each group.

This study employed a blind method; the subjects were not informed of their group assignments from the time of enrollment until the end of the follow-up. The surgery was performed by the same group of surgeons, while postoperative management and follow-up data collection were handled by another dedicated physician. The data collection personnel were unaware of the group assignments of the subjects.

### 2.3 Anesthesia and surgical methods

#### 2.3.1 Key surgical instruments

Energy instruments: high-frequency electric knife, hand-controlled electric hook, ultrasound knife. Bronchial resection instruments: cutting stapler and corresponding anastomosis staples, 4.0 silk thread, knot pusher, endoscopic tissue scissors. Lymph node biopsy forceps, incision protector, single and double-joint endoscopic grasping forceps and dissection forceps, as well as general instruments necessary for endoscopic surgery.

#### 2.3.2 Preoperative preparation and anesthesia methods

In this study, all patients underwent standardized preoperative evaluation and preparation to eliminate biases caused by differences in preoperative medication on the study outcomes.

##### 2.3.2.1 Airway preparation

For patients meeting the inclusion criteria, smoking cessation for two weeks prior to surgery was required, along with regular use of inhaled corticosteroids (such as budesonide) via nebulization for three days leading up to the surgery.

##### 2.3.2.2 Cough management

Patients who had upper respiratory infections, pulmonary infections, or other cough symptoms within two weeks prior to surgery were excluded from this study to eliminate any interference with the assessment of postoperative cough.

##### 2.3.2.3 Routine preoperative medication

Thirty minutes before anesthesia, routine intramuscular administration of an anticholinergic agent (glycopyrrolate, 0.01 mg/kg) was conducted. The use of this medication aims to suppress glandular secretion and keep the airway dry while providing some bronchial dilation, which helps stabilize the airway during surgery.

##### 2.3.2.4 Record of co-medications

We meticulously recorded all preoperatively used chronic medications in all patients, particularly angiotensin-converting enzyme (ACE) inhibitors. These medications are known cough-inducing agents, and patients taking these medications were switched to alternative antihypertensives (such as angiotensin II receptor antagonists) at least one week prior to surgery.

##### 2.3.2.5 Anesthesia protocol

All patients received a standardized general anesthesia protocol. Anesthesia induction involved the use of midazolam (0.03-0.05 mg/kg), propofol (1.5-2.5 mg/kg), sufentanil (0.4-0.6 μg/kg), and cisatracurium (0.15-0.2 mg/kg). Once complete muscle relaxation was achieved, a same experienced anesthesiologist performed double-lumen bronchial intubation for single-lung ventilation, using a fiberoptic bronchoscope to confirm the accurate positioning of the tube.

##### 2.3.2.6 Airway management during anesthesia

Before and during single-lung ventilation, regular suctioning of secretions from the airway and double-lumen tube was performed using a fiberoptic bronchoscope to maintain airway cleanliness and avoid secretions triggering postoperative cough.

#### 2.3.3 Surgical implementation

Prior to the surgery, both groups of patients underwent routine 3D-CTBA to identify the anatomical structures of the lung hilum and assess the feasibility of segmental resection. General anesthesia was administered through double-lumen tracheal intubation, and a single-port thoracoscopic segmental resection was performed. The anatomical structures of the hilum were dissected, the segmental blood vessels and bronchi were severed, and the intersegmental planes were identified using a ventilation collapse method before incision with the stapler. Selective lymph node sampling was carried out, which included the excision of lymph nodes 12 and 13, along with segmental resections of the right upper lobe (4R and 10), right lower lobe (11s and 11i), left upper lobe (5, 6, and 10), left lingular segment (11), and left lower lobe (10 and 11). All surgeries were performed by the same group of senior surgeons to minimize biases arising from differences in surgical techniques. The specific procedures for each group were as follows:

##### 2.3.3.1 Experimental group

**Figure 1:**
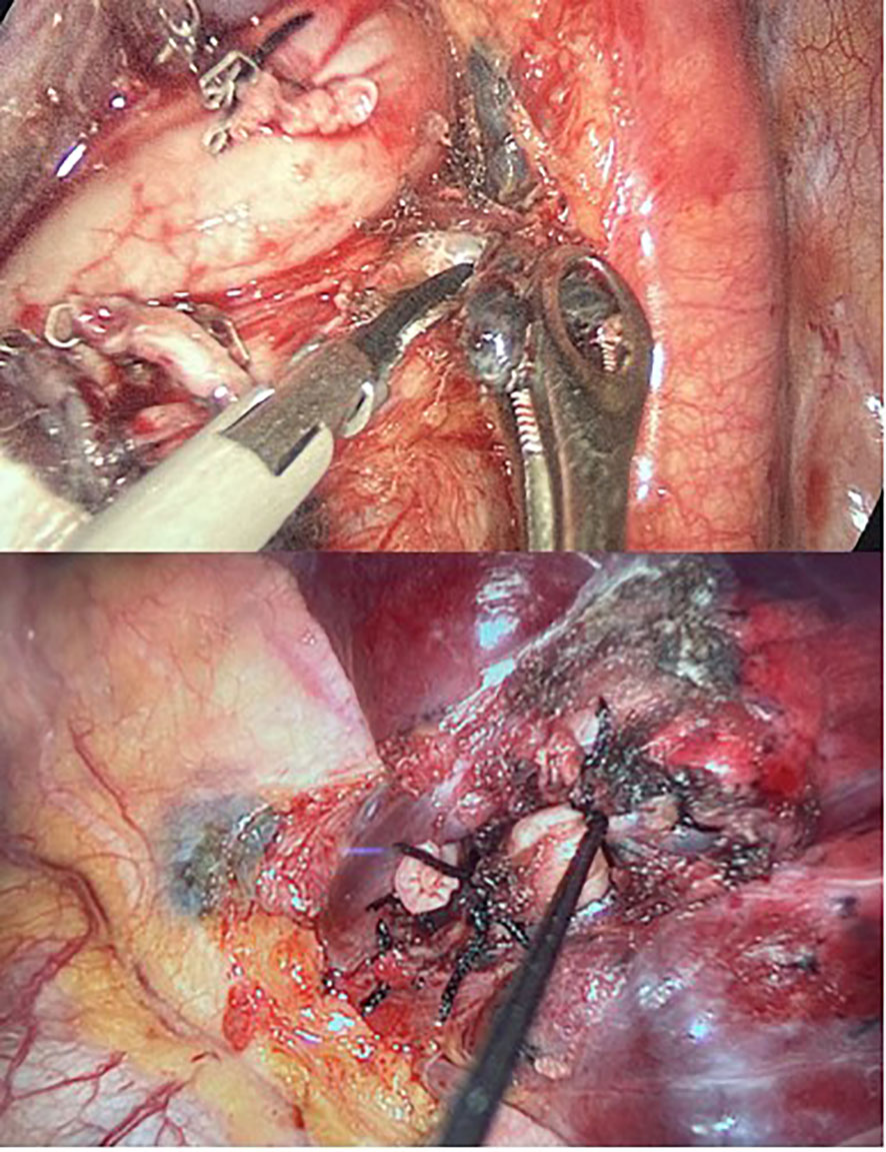
Ultrasonic dissection + double silk ligation.

Ultrasonic dissection + double silk ligation: Under the premise of ensuring safety, the use of the ultrasonic scalpel should be prioritized for segmental hilar dissection and lymph node sampling, minimizing the use of electrocautery hooks. For the proximal bronchus of the lung segment, double ligation was performed with a 4.0 silk thread as close to the root as possible, followed by single ligation and transection at the distal end. For the pulmonary segmental vessels, double ligation was performed at the proximal end with a 1.0 silk thread, followed by single ligation and transection at the distal end (**Figure 1**).

##### 2.3.3.2 Control group

**Figure 2:**
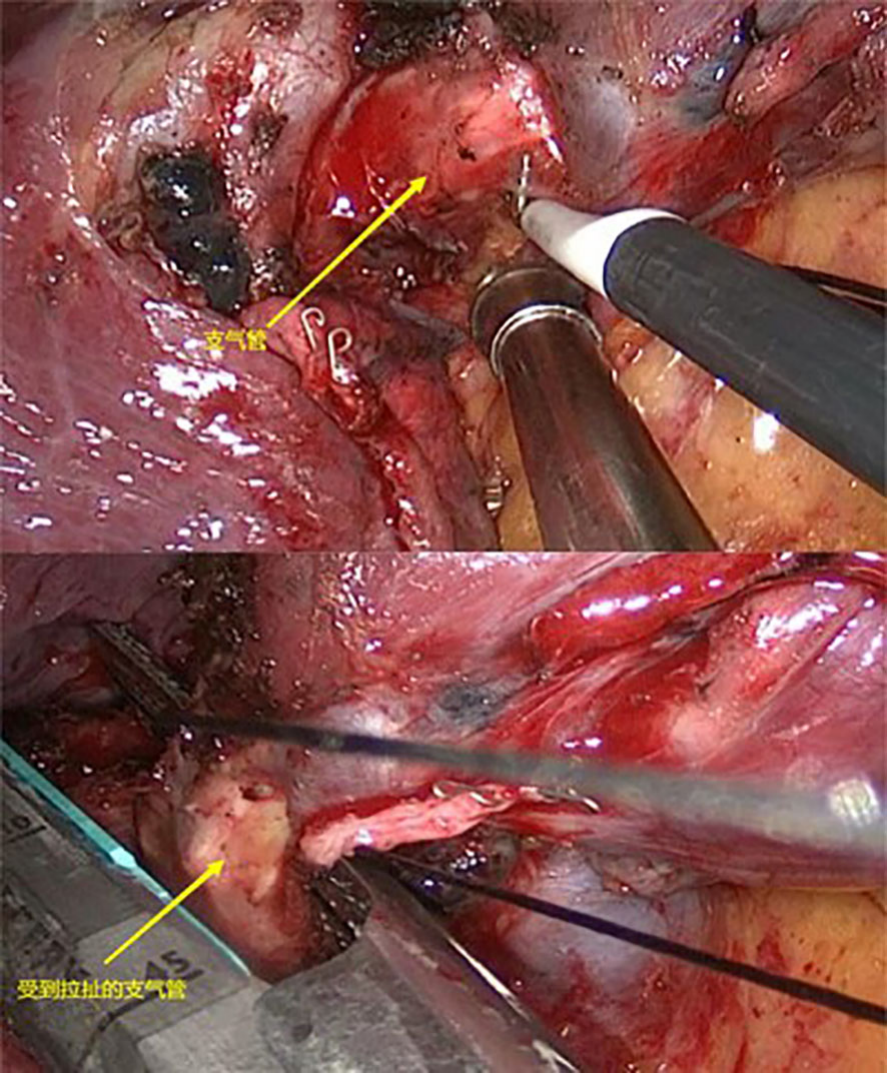
Electric coagulation hook dissection + stapler transection.

Electric coagulation hook dissection + stapler transection: Under the premise of ensuring safety, the use of electrocautery hooks should be prioritized for segmental hilar dissection and lymph node sampling, minimizing the use of ultrasonic scalpels. The bronchus of the lung segment was transected using an endoscopic stapler as close to the root as possible. The segmental vessels were also transected using the stapler (**Figure 2**).

### 2.4 Observation indicators

#### 2.4.1 Postoperative cough assessment

The Mandarin Chinese version of the Leicester Cough Questionnaire (LCQ-MC) and the Visual Analogue Scale (VAS) are used to evaluate postoperative quality of life and the severity of cough symptoms. The effectiveness of the study on postoperative airway inflammation indicators was assessed through white blood cell count and high-sensitivity C-reactive protein in blood samples. Follow-up assessments are conducted in person or via phone at 2 weeks, 4 weeks, and 8 weeks post-surgery.

#### 2.4.2 Intraoperative data

Surgical duration, intraoperative blood loss, number of lymph nodes dissected and their groups.

#### 2.4.3 Postoperative data

Duration of chest tube placement, total amount of thoracic drainage, and length of postoperative hospital stay.

#### 2.4.4 Incidence of severe complications

The rates of death, respiratory failure, bronchopleural fistula, secondary surgery for hemothorax, chylothorax, and other severe complications.

### 2.5 Statistical analysis

Data is processed using SPSS version 26.0. Continuous variables are expressed as means and analyzed using the t-test; categorical variables are expressed as frequency/percentage (%) and analyzed using chi-square tests. P <0.05 was considered statistically significant.

### 2.6 Ethics review

The study strictly adheres to the relevant regulations of the Declaration of Helsinki and the Ethical Review Measures for Biomedical Research Involving Humans. It has been approved by the Ethics Committee of Tongling Municipal People’s Hospital. Patient participation in the study is entirely voluntary, and they can withdraw at any time without affecting their routine treatment. All patient information is kept strictly confidential, and the research data will be used solely for academic purposes.

## 3 Results

### 3.1 General Information

There were no statistically significant differences (P > 0.05) in baseline characteristics between the two groups of patients regarding gender, age, smoking history, average smoking duration for smokers, surgical resection of lung segments, and pathological nature of nodules. See **Table 1**.

**Table 1:** Comparison of baseline data between the two groups of patients.

Variables	Experimental group (n=50)	Control group (n=50)	t/χ2 value	P value
Sex			0.041	0.840
Male [n (%)]	22 (44%)	21 (42%)		
Female [n (%)]	28 (56%)	29 (58%)		
Age (, years)	60.80 ± 9.04	58.50 ± 10.63	1.165	0.247
Smoking history [n (%)]	17 (34%)	19 (38%)	0.174	0.677
Average smoking duration for smokers (, years)	19.82 ± 10.74	21.05 ± 11.07	0.578	0.565
Lung Segment Location			2.437	0.487
Right Upper Lobe	18 (36%)	18 (36%)		
Right Lower Lobe	14 (28%)	11 (22%)		
Left Upper Lobe	10 (20%)	16 (32%)		
Left Lower Lobe	8 (16%)	5 (10%)		
Nodule Pathological Nature:			1.268	0.530
Carcinoma *in situ* and non-cancerous lesions	22 (44%)	26 (52%)		
Minimally invasive cancer	17 (34%)	10 (20%)		
Invasive cancer	11 (22%)	14 (28%)		

### 3.2 Postoperative cough assessment

In this study, patients in the modified bronchial protection measures group (experimental group) and the traditional bronchial protection measures group (control group) were followed up for 8 weeks, focusing on evaluating the intensity of postoperative cough symptoms and quality of life. The results indicated that the modified measures had significant advantages in reducing postoperative cough symptoms and improving patients’ quality of life.

The project team assessed the intensity of cough symptoms using the Visual Analogue Scale (VAS), where lower scores indicate lesser intensity of cough symptoms. **Table 2** presents a comparison of the VAS scores for cough symptoms between the two groups of patients at 2 weeks, 4 weeks, and 8 weeks post-surgery.

**Table 2:** Comparison of postoperative cough VAS scores between the experimental group and the control group.

Time point	Experimental group (mean, ± s)	Control group (mean, ± s)	t value	P value
Postoperative 2 Weeks	2.86 ± 2.23	3.76 ± 2.27	1.998	0.048
Postoperative 4 Weeks	1.50 ± 1.34	2.38 ± 1.52	3.063	0.003
Postoperative 8 Weeks	0.76 ± 0.89	1.28 ± 1.13	2.559	0.012

The data shows that the experimental group had significantly lower cough VAS scores at all follow-up time points compared to the control group. At 2 weeks post-surgery, the experimental group’s cough VAS score was 2.86, while the control group’s score was 3.76 (P<0.05). This difference persisted at 4 weeks and 8 weeks post-surgery, indicating that the modified measures have a sustained effect in reducing postoperative cough symptoms.

**Figure 3:**
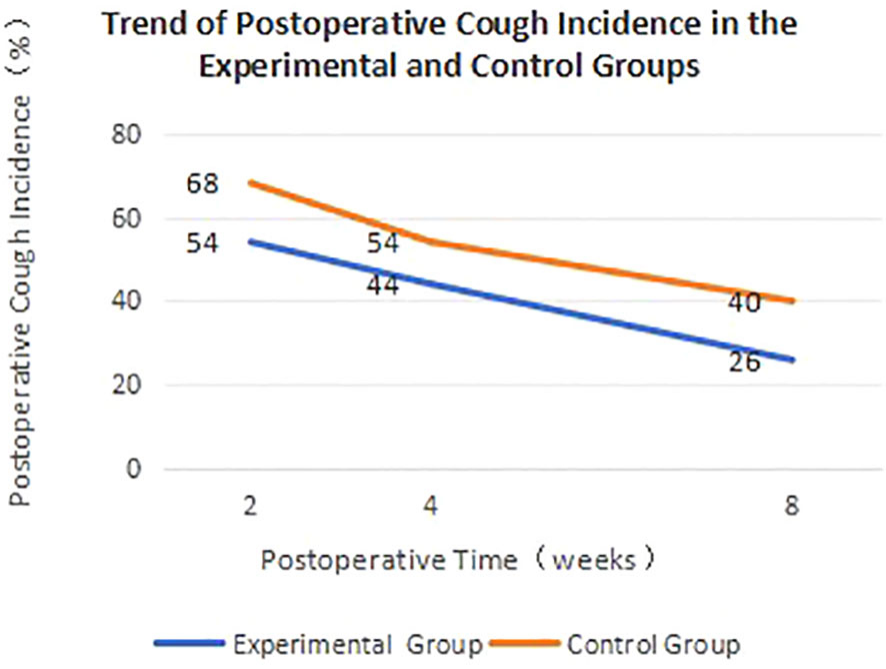
Trend of postoperative cough incidence in the experimental and control groups.

Postoperative cough incidence is one of the key indicators for evaluating surgical outcomes. Patients with a VAS score >1 for cough are considered to still have postoperative cough. To visually demonstrate the changes in postoperative cough incidence between the two groups, we have plotted **Figure 3**.

**Figure 3** clearly illustrates the trend of postoperative cough incidence over time for both groups of patients. The cough incidence in the experimental group was not only lower at each time point compared to the control group, but it also decreased at a faster rate, indicating that the modified measures can accelerate the recovery process for patients.

We used the Mandarin Chinese version of the Leicester Cough Questionnaire (LCQ-MC) to assess the patients’ postoperative quality of life, with scores ranging from 3 to 21, where higher scores indicate less impact from postoperative cough and higher quality of life. Preoperative and postoperative white blood cell counts and high-sensitivity C-reactive protein were collected from patients as inflammatory indicators to assess the level of airway inflammation. **Table 3** shows the LCQ-MC scores of the two groups of patients at different time points, while **Table 4** presents the inflammation levels of the two groups at different time points.

**Table 3:** Comparison of postoperative LCQ-MC scores between the experimental group and the control group.

Time point	Experimental group (score, ± s)	Control group (score, ± s)	t value	P value
Postoperative 2 Weeks	14.60 ± 1.99	13.36 ± 1.30	3.724	<0.001
Postoperative 4 Weeks	16.72 ± 1.28	15.43 ± 1.25	5.095	<0.001
Postoperative 8 Weeks	18.62 ± 1.02	17.56 ± 0.87	5.575	<0.001

**Table 4:** Comparison of postoperative inflammatory marker levels between the experimental group and the control group.

Time point	Experimental group	Control group	t value	P value
White blood cell count.(*10^9^/L, ±)				
Three days before the surgery.	5.43 ± 1.57	5.17 ± 1.31	0.902	0.370
Three days after surgery.	9.75 ± 3.15	13.37 ± 4.66	4.562	<0.001
Postoperative 2 Weeks	8.01 ± 2.29	8.96 ± 3.52	1.608	0.111
Postoperative 4 Weeks	6.25 ± 2.60	6.70 ± 2.03	0.962	0.338
Postoperative 8 Weeks	5.92 ± 1.70	5.92 ± 2.33	0.008	0.993
High-sensitivity C-reactive protein.(mg/L, ±)				
Three days before the surgery.	2.12 ± 2.74	2.31 ± 2.44	0.375	0.708
Three days after surgery.	26.52 ± 15.40	36.36 ± 29.53	2.090	0.039
Postoperative 2 Weeks	13.19 ± 7.39	18.52 ± 17.08	2.025	0.046
Postoperative 4 Weeks	7.53 ± 5.74	7.68 ± 6.38	0.125	0.901
Postoperative 8 Weeks	3.67 ± 3.44	3.77 ± 3.95	0.136	0.892

The data shows that the experimental group had significantly higher LCQ-MC scores at all follow-up time points compared to the control group (P<0.05). This result further confirms the superiority of the modified measures in reducing the impact of postoperative cough and improving quality of life.

The data show that there were no significant differences in preoperative inflammatory markers between the two groups (P>0.05). On the third day after surgery, the white blood cell count and high-sensitivity C-reactive protein levels in the experimental group were significantly lower than those in the control group (P<0.05). Two weeks postoperatively, the level of high-sensitivity C-reactive protein was significantly lower than that in the control group (P<0.05), while the white blood cell count did not reach statistical significance (P>0.05), although the average was lower than that of the control group. At four and eight weeks postoperatively, the white blood cell count and high-sensitivity C-reactive protein levels were slightly lower than those in the control group, but the differences did not reach statistical significance (P>0.05). These results indicate that the level of airway inflammation may play a certain role in acute cough after surgery, but it is not a core factor in the occurrence of subacute and chronic postoperative cough.

### 3.3 Intraoperative data comparison

To comprehensively assess the safety and effectiveness of the modified intraoperative bronchial protection measures, this study conducted a detailed comparison and analysis of the intraoperative-related indicators between the experimental and control groups. The main comparative indicators included intraoperative blood loss, surgical duration, and the number of lymph nodes dissected (**Table 5**).

**Table 5:** Comparison of intraoperative surgical indicators.

Variables	Experimental group (n=50)	Control group (n=50)	t value	P value
Intraoperative Blood Loss (mL)	26.50 ± 11.48	46 ± 85.23	1.603	0.112
surgical duration (min)	2.32 ± 0.68	2.42 ± 1.03	0.569	0.571
Number of Mediastinal Lymph Nodes Dissected	3.52 ± 3.56	3.46 ± 4.07	0.078	0.938

The data shows that the average intraoperative blood loss in the experimental group did not reach statistical significance (P>0.05), but the average was significantly lower than that of the control group. The average surgical duration in the experimental group was slightly shorter than that in the control group, but the difference was not statistically significant (P>0.05). There was no significant difference in the average number of lymph nodes dissected between the two groups (P>0.05).

### 3.4 Postoperative data comparison

To assess the impact of the modified intraoperative bronchial protection measures on postoperative recovery, this study compared several postoperative indicators between the experimental and control groups. The main comparative indicators included total drainage volume within 3 days post-surgery, duration of chest tube placement, length of postoperative hospital stay, and incidence of postoperative complications (**Table 6**).

**Table 6:** Comparison of postoperative recovery.

Variables	Experimental group (n=50)	Control group(n=50)	t value	P value
Volume of Drainage on Postoperative (mL)	583.80 ± 243.52	547.6 ± 308.22	0.652	0.516
Duration of Chest Tube Placement (d)	4.00 ± 1.47	4.26 ± 1.90	0.764	0.447
Postoperative Hospital Stay Duration (d)	5.80 ± 1.92	6.34 ± 2.10	1.344	0.182
Postoperative ICU Admission [n (%)]	3(6%)	4(8%)	0.514	0.695
ICU Length of Stay (d)	3.67 ± 0.748	3.75 ± 1.055	0.656	0.513
Postoperative Complications Occurrence [n (%)]	5(10%)	8(16%)	0.796	0.372
Pulmonary Infection [n (%)]	1(2%)	1(2%)	0.000	1.000
Pneumothorax [n (%)]	1(2%)	3(6%)	1.042	0.307
Atelectasis [n (%)]	0(0%)	1(2%)	1.010	0.315
Arrhythmia [n (%)]	3(6%)	4(8%)	0.154	0.695

The data shows that there was no significant difference in the total drainage volume post-surgery between the two groups (P>0.05). The duration of chest tube placement and length of postoperative hospital stay in the experimental group were slightly shorter than those in the control group, but this difference was not statistically significant (P>0.05).

The occurrence of postoperative complications in the two groups is shown in **Table 6**. Neither group had cases of respiratory failure, bronchopleural fistula, reoperation due to hemothorax, or chylothorax, and there were no perioperative deaths. The overall complication rate in the experimental group was slightly lower than that in the control group, but this difference was not statistically significant (P > 0.05). Postoperative complications in both groups improved after symptomatic treatment, allowing for discharge.

### 3.5 Multifactorial analysis of factors affecting postoperative chronic cough

To demonstrate that our intervention has an independent effect, we used the postoperative chronic cough LCQ-MC score as the dependent variable and other factors influencing the occurrence of postoperative cough as covariates for the logistic regression analysis (**Table 7**).

**Table 7:** Multifactorial analysis of factors affecting postoperative chronic cough.

Influencing factors	B	95%CI	Standardized regression coefficient	Standard error	Statistic value	P value
Modified measures	-1.129	(-1.504,-0.754)	-0.524	0.189	-5.978	<0.001
Smoking history	0.169	(-0.773,1.111)	0.075	0.474	0.356	0.723
Average smoking duration for smokers	0.008	(-0.034,0.051)	0.083	0.021	0.386	0.7
Sex	-0.26	(-0.642,0.122)	-0.12	0.192	-1.354	0.179
Intraoperative Blood Loss	0.001	(-0.002,0.005)	0.085	0.002	0.899	0.371
surgical duration	-0.023	(-0.245,0.199)	-0.019	0.112	-0.209	0.835
Number of Mediastinal Lymph Nodes Dissected	-0.016	(-0.066,0.035)	-0.055	0.025	-0.619	0.537

The results indicate that after controlling for potential confounding variables, the modified measures we implemented remain independent protective factors for reducing the risk and severity of postoperative cough (B = -1.129, 95% CI: -1.504 to -0.754, p < 0.001).

## 4 Discussion

This study aims to evaluate the use of suture ligatures and the avoidance of thermal injury to comprehensively examine their potential impact on a range of clinical outcomes in patients following pulmonary segmentectomy. In addition to traditional postoperative complications such as those related to intraoperative safety, pneumothorax, atelectasis, pulmonary infection, and bleeding, we specifically included postoperative cough symptoms in the core assessment framework. The results indicate that the modified measures significantly alleviate postoperative cough symptoms, enhance patients’ quality of life, and promote postoperative recovery. These findings not only hold important clinical significance but also provide new insights for optimizing perioperative management in video-assisted thoracoscopic pulmonary segmentectomy.

**Figure 4:**
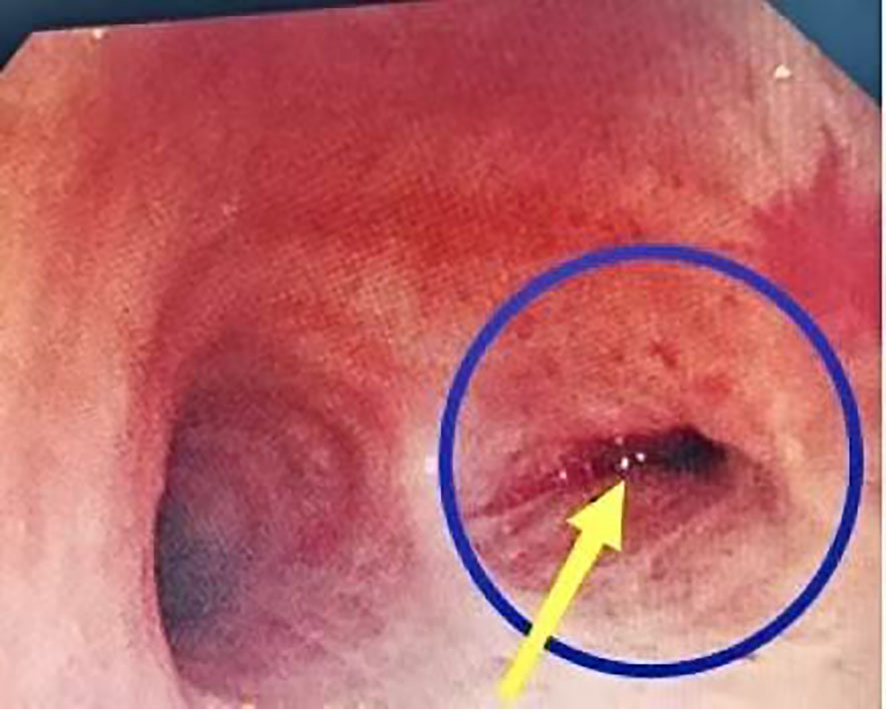
Foreign body in the form of sutures visible in bronchoscopy.

The occurrence of cough after pulmonary resection may be related to excessive stimulation of the trachea and impaired pathways of cough neurons. Based on the surgical operations of segmentectomy and the distribution characteristics of cough receptors and vagal nerve pulmonary plexus, the proposed mechanisms may include: First, the anatomical procedure damages the walls of the trachea and bronchi (5, 6); second, the bronchial walls may be subjected to abnormal mechanical traction or directly exposed to various chemical irritants, leading to abnormal excitation of the cough reflex (9, 10); third, damage to the afferent fibers responsible for coughing during the anatomical procedure may increase their sensitivity during the repair process (11); fourth, stimulation of the bronchi by surgical scars, sutures, and other foreign materials may contribute to the occurrence of postoperative cough (12–13) (**Figure 4**).

It is important to recognize that the occurrence of postoperative cough is a multifactorial process that may be influenced by various confounding factors. To accurately assess the independent effect of the “improved intraoperative bronchial protection measures,” we ensured no significant baseline differences among the patient groups through strict inclusion and exclusion criteria. Additionally, we implemented standardized anesthesia protocols, postoperative analgesia, and lung expansion strategies to minimize the interference caused by anesthetic drugs, intubation injuries, pain, and atelectasis. We also kept detailed records and comparisons of complications such as postoperative air leaks. Subsequent multivariate regression analysis revealed that, after adjusting for the aforementioned potential confounders, the modified bronchial protection measures remained an independent protective factor for reducing the risk and severity of postoperative cough. This indicates that the intervention has a positive effect on alleviating cough after video-assisted thoracoscopic pulmonary segmentectomy, independent of other common factors.

The causes and influencing factors of postoperative cough are quite complex. This study focuses on the specific aspect of “bronchial handling techniques” to explore and validate the specific and intervenable factor of “the energy and physical damage to the bronchi caused by energy devices and staplers.” The “modified measures” in this study are designed to maximize the isolation and reduction of this specific source of damage (14, 15). After controlling for other key factors, we aim to determine whether different bronchial handling methods still have an independent association with the incidence and duration of cough. Based on the potential mechanisms of postoperative cough occurrence mentioned above, the research team proposed and implemented improved bronchial protection measures, which offer the following advantages compared to traditional surgical methods: (1) Suture ligation reduces mechanical damage to the bronchial stump and avoids excessive traction and foreign body residue that may result from cutting staplers; (2) The use of an ultrasonic scalpel instead of an electrocautery hook to separate tissues reduces thermal and electrical damage to bronchial nerve tissues; (3) Refined surgical techniques reduce irritation to the bronchial mucosa and decrease the intensity of postoperative inflammatory responses. These factors work together to effectively reduce the sensitivity of the postoperative cough reflex.

Many patients experience postoperative cough without clear signs of respiratory or pulmonary infection, or pneumothorax, often presenting as unexplained dry coughs. Conventional drug treatments are often ineffective (16). The “Diagnosis and Treatment Guidelines for Cough” published by the Respiratory Disease Branch of the Chinese Medical Association classifies coughs into acute cough (<3 weeks), subacute cough (3–8 weeks), and chronic cough (>8 weeks), with subacute and chronic coughs being more common after lung surgery (17). The “Primary Care Guidelines for Cough” issued by the Chinese Medical Association categorizes coughs into dry and productive coughs, recommending a threshold of less than 10 mL of sputum per day as the standard for dry cough (18). Therefore, the project team chose to evaluate postoperative cough at 2 weeks, 4 weeks, and 8 weeks after surgery, with stratified comparisons of the incidence of acute cough (<3 weeks), subacute cough (3–8 weeks), and chronic cough (>8 weeks), attempting to identify the patterns of occurrence.

The modified measures significantly reduced the incidence of postoperative cough. During the follow-up at 2 weeks, 4 weeks, and 8 weeks post-surgery, the incidence of cough in the experimental group was consistently significantly lower than in the control group. This result contrasts sharply with the 25%-50% incidence of cough reported in previous studies using traditional surgical methods (19). It also shows an improvement over the 66.1% postoperative cough incidence at 4 weeks reported by Chen et al. (20) using traditional cutting staplers. At the same time, the study by Zhang et al. (21) found that two-thirds of patients still had cough symptoms higher than pre-surgery levels at discharge, and even at 4 weeks post-surgery, the incidence of cough was still over 50%. This suggests that the modified measures proposed in this study have significant advantages in reducing the incidence of postoperative cough.

The modified measures significantly improved the intensity of postoperative cough symptoms and quality of life. Research data show that the experimental group had significantly lower scores on the Mandarin Chinese version of the Leicester Cough Questionnaire (LCQ-MC) at all follow-up time points compared to the control group. This result surpasses the cough scores reported by Xie et al. (22) after implementing standardized surgical procedures (17.31 ± 1.19 at 8 weeks post-surgery and 18.29 ± 0.74 at 10 weeks post-surgery).

It is noteworthy that the improvement in cough symptoms in the modified measures group occurred at a faster rate, which may be related to the reduced tissue damage associated with silk ligation and the use of ultrasonic knives. According to previous studies, an increase in LCQ-MC scores reflects a significant improvement in patients’ cough symptoms (23). In this study, the experimental group’s LCQ-MC scores were, on average, 1.24, 1.29, and 1.06 points higher than those of the control group at 2 weeks, 4 weeks, and 8 weeks post-surgery, respectively, indicating that the modified measures can continuously and effectively alleviate patients’ cough symptoms. This improvement in symptoms not only enhances patient comfort but may also reduce psychological anxiety caused by surgery or tumors (24).The results show that there were no significant differences in the timing of drainage tube removal between the two groups. Even in the early stages, the implementation of the measures in this study was able to reduce the incidence and severity of cough scores postoperatively, which was statistically significant. However, according to the results of the postoperative cough VAS scores, the impact of related factors associated with energy devices and staplers at two weeks postoperatively (P = 0.048, <0.05) was not as pronounced as at four weeks (P = 0.003, <0.05) and eight weeks (P = 0.012, <0.05) postoperatively. This result may indicate that our improvement measures have limited effects on early postoperative cough, primarily reducing the occurrence and severity of subacute and chronic postoperative coughs.

From the perspective of intraoperative indicators, the modified measures demonstrated good safety and effectiveness. Intraoperative blood loss is one of the key indicators for assessing surgical safety. Experimental results indicate that the improved bronchial protection measures not only did not increase the risk of surgical bleeding but, to some extent, reduced the amount of intraoperative bleeding. This demonstrates the advantages of the modified measures in controlling intraoperative bleeding, which may be related to the precise cutting and immediate hemostatic effects of the ultrasonic scalpel (25). Compared to other research reports on intraoperative blood loss in segmentectomy, this study shows a slight advantage in reducing intraoperative blood loss (26).Surgical time is a key indicator for assessing surgical efficiency. The results showed that the average surgical time in the experimental group was 2.32 hours, which was 0.1 hour shorter than the control group, similar to the surgical time reported in previous studies on segmentectomy (27). Although the difference in surgical time between the two groups did not reach statistical significance (P>0.05), as the modified measures are applied more effectively and the process is further optimized, the surgical time can be shortened even more. This shows that the method achieved refined handling of the bronchus, highlighting the efficiency of the modified measures. The completeness of mediastinal lymph node dissection is an important indicator for evaluating lung cancer staging, preventing recurrence, and improving patient survival. The data show that the number of lymph node dissections in pulmonary segmentectomy in this study is similar to that in previous studies (28). This result indicates that the modified bronchial protection measures do not interfere with routine lymph node dissection and can ensure the thoroughness of the surgery.

Postoperative-related indicators further confirm the clinical value of the modified measures. The experimental group showed a significant reduction in postoperative drainage volume, shorter drainage tube placement time, and a slight reduction in pneumothorax duration, all of which are important indicators for assessing the degree of surgical trauma and recovery speed. The decrease in drainage volume and the shortening of drainage time may be related to the reduced tissue damage and inflammatory response caused by the modified measures (29). The reduction in pneumothorax duration may be attributed to the more refined bronchial handling technique, which minimized lung tissue damage (30). These improvements not only accelerate patient recovery but may also lower hospitalization costs and enhance the efficiency of medical resource utilization.

Although this study did not observe significant differences in the incidence of postoperative complications, the experimental group had a lower overall complication rate (4% vs 8%), which is a trend worth noting. Particularly, the incidence of pulmonary infections and atelectasis was lower in the experimental group compared to the control group. This may be related to the alleviation of cough symptoms, as effective coughing helps clear airway secretions and prevent pulmonary complications (31). However, due to the limitation of sample size, this difference did not reach statistical significance. Future large-scale studies may provide more conclusive evidence.

This study provides an effective new method to reduce postoperative cough, a common symptom following thoracoscopic segmentectomy. The application of modified measures is expected to significantly improve this clinical issue. The results support the practice of the “precision medicine” concept in minimally invasive thoracic surgery. By refining the handling of bronchial stumps and surrounding tissues, the surgery minimizes trauma to the greatest extent possible, reflecting a shift from passive prevention to active protection (32). The findings of this study offer important insights for the development of perioperative management strategies. The modified measures not only optimized the surgical process but also simplified postoperative management by reducing postoperative cough and related complications. This “front-end” optimization approach may be more effective than traditional symptomatic postoperative treatment and aligns better with modern surgery’s pursuit of comprehensive management and rapid recovery (33).

However, this study also has some limitations. Firstly, as a single-center prospective cohort study, the patient population, the surgical team’s technical background, and perioperative management strategies exhibit a certain degree of homogeneity, which may limit the generalizability of the study results to other medical environments. Future research should further validate the universal applicability of using suture ligatures as a replacement for staplers and controlling thermal energy on postoperative outcomes in pulmonary segmentectomy through multi-center, large-sample randomized controlled trials in different technical settings and patient populations. Secondly, the follow-up period in this study was set to 8 weeks postoperatively, but it could not assess the long-term effects of the modified measures on patient prognosis. Future studies could consider extending the follow-up period to evaluate the long-term effects on outcomes such as pulmonary function recovery, exercise endurance, chronic pain, or quality of life. Moreover, this study primarily focuses on the improvement of clinical symptoms and quality of life, lacking in-depth exploration of potential mechanisms, such as levels of inflammatory factors and the integrity of neuronal pathways. In future prospective studies, we plan to collect induced sputum or bronchial lavage fluid to directly measure biological markers related to cough, such as IL-6, IL-8, and TNF-α, to reflect the effectiveness of the study. Additionally, although we made efforts to control confounding factors, due to the observational nature of the study design, we cannot completely eliminate the effects of selection bias and residual confounding. For instance, factors such as baseline characteristics, lesion location, and extent may have interfered with the outcomes. Future research could adopt propensity score matching or stratified analysis to enhance comparability between groups. In conclusion, while this study suggests that suture ligation combined with thermal energy control may benefit early recovery after pulmonary segmentectomy, its generalizability, long-term effects, and underlying mechanisms still require further high-quality research for validation and deeper exploration.

## 5 Conclusion

In conclusion, Our study suggests that using suture ligatures as a replacement for staplers and minimizing thermal injury to the hilar structures may have a positive impact on postoperative clinical outcomes, particularly the severity and duration of postoperative cough, leading to a significant improvement in patients’ quality of life. It is a safe and effective intraoperative management approach. This innovative technique provides new possibilities for improving the surgical outcomes and prognosis of patients undergoing thoracoscopic segmentectomy. Future research should focus on further optimizing technical details, exploring its long-term effects, and validating its effectiveness in a broader population. Additionally, combining this technique with other perioperative optimization strategies is expected to further enhance the overall therapeutic effect of thoracoscopic segmentectomy, providing greater clinical benefits for patients.

## Data Availability

The original contributions presented in the study are included in the article/supplementary material. Further inquiries can be directed to the corresponding author.
